# Genome Sequence of *Bacillus pumilus* Strain Bonn, Isolated from an Anthrax-Like Necrotic Skin Infection Site of a Child

**DOI:** 10.1128/genomeA.01741-15

**Published:** 2016-02-11

**Authors:** Gregor Grass, Gabriele Bierbaum, Ernst Molitor, Natascha Götte, Markus Antwerpen

**Affiliations:** a Bundeswehr Institute of Microbiology, Munich, Germany; b Institute of Medical Microbiology, Immunology and Parasitology (IMMIP), University of Bonn, Bonn, Germany; c Pediatric Surgery, Lohmar, Germany

## Abstract

We report the draft genome sequence of *Bacillus pumilus* strain Bonn associated with human skin infection. *B. pumilus* Bonn was isolated from a carbuncle-like necrotic site, resembling cutaneous anthrax, on the back of the hand of a 10-year-old child.

## GENOME ANNOUNCEMENT

Typically, *Bacillus pumilus* is an inhabitant of soil environments, but symbiotic or plant pathogenic strains or probiotic strains of animals are known (1). Some cases have been described where *B. pumilus* was associated with infections in human-like cutaneous lesions, catheter infections, or severe sepsis of neonatal infants (2–4).

*B. pumilus* strain Bonn was isolated from a 10-year-old boy in 2011. The patient exhibited a prominent inflammation on the back of his right hand with a central necrosis of about 4-cm diameter (E. Molitor, unpublished data). After surgical debridement, the necrotic lesion enlarged. Therefore, a swab was taken and cultured from the edge of the lesion yielding *B. pumilus* among normal skin flora. The lesion resolved upon treatment with amoxicillin-sulbactam. Similar case descriptions have been made for earlier *B. pumilus* infections (4). These authors suggested that *B. pumilus* produced dermonecrotic factors causing lesions (4).

Here we present, to the best of our knowledge, the first genome sequence reported of a *B. pumilus* strain associated with human necrotic skin infection. In order to identify virulence factors it will be interesting to compare the genome of strain Bonn with the previously characterized *B. pumilus* strains exhibiting similar clinical manifestations (4).

Whole-genome shotgun (WGS) sequencing of *B. pumilus* Bonn was performed by Ion Torrent sequencing technology (Ion Torrent Systems Inc, USA). For the WGS library, 1,705,145 reads with a total of 460 Mb were generated. Using Newbler version 2.6 software (Roche Diagnostics, Darmstadt, Germany), 99.57% of the reads were *de-novo* assembled, resulting in 14 contigs larger than 500 bp, which were used for further analysis. The G+C content was calculated using an in-house Python script.

The total length of the genome shotgun sequence of *B. pumilus* Bonn was 3,662,762 bp with a 215-fold coverage, and the mean G+C content was 41.5%. No plasmids were identified. For initial annotation, assembled contigs were submitted to the RAST server (5).

The *B. pumilus* Bonn draft genome encodes 3,544 putative coding sequences. Based on the coverage we estimate that the genome sequence featured five copies of the 16S rRNA, the 5S rRNAs, and the 23S rRNA; 68 tRNA loci were clearly identified. The most similar strain to strain Bonn in databases is *B. Pumilus* strain SAFR-032 (GenBank accession no. NC_009848.1) with an average sequence identity of 95.7%. Contig 12 of *B. pumilus* Bonn encodes a probable prophage which has not been previously described in any other *Bacillus* species and which comprises mostly hypothetical genes with no significant similarity hits. In addition, no *B. anthracis*-like virulence factor genes potentially involved in its clinical manifestation, such as those for lethal factor (*lef*), edema factor (*cya*), protective antigen (*pagA*), or for a poly-gamma-D-glutamyl-capsule were identified in the genome of *B. pumilus* Bonn. A *fosB*-like gene was identified as possibly responsible for the previously observed fosfomycin resistance (E. Molitor, unpublished).

The initial clinical manifestation is reminiscent of a *Pustula maligna*, to which the assumption of toxin production would match. Skin infections by *B. pumilus* with symptoms similar to cutaneous anthrax have been described elsewhere (4) and should not be generally interpreted as contamination of the culture.

### Nucleotide sequence accession numbers.

This whole-genome shotgun project has been deposited at DDBJ/EMBL/GenBank under accession number LNCN00000000. The version described in this paper is the first version, LNCN01000000.
